# Metabolomic Approaches to Studying the Response to Drought Stress in Corn (*Zea mays*) Cobs

**DOI:** 10.3390/metabo11070438

**Published:** 2021-07-03

**Authors:** Isabella Gaffney, Jonathan Brett Sallach, Julie Wilson, Edmund Bergström, Jane Thomas-Oates

**Affiliations:** 1 Department of Chemistry, University of York, York YO10 5DD, UK; irg504@york.ac.uk (I.G.); ed.bergstrom@york.ac.uk (E.B.); jane.thomas-oates@york.ac.uk (J.T.-O.); 2 Department of Environment and Geography, University of York, York YO10 5NG, UK; 3 Department of Mathematics, University of York, York YO10 5DD, UK; julie.wilson@york.ac.uk; 4 Centre of Excellence in Mass Spectrometry, University of York, York YO10 5DD, UK

**Keywords:** maize, metabolomics, LC–MS, high-resolution mass spectrometry, abiotic stress, climate change

## Abstract

Metabolomics is a technique that allows for the evaluation of the entire extractable chemical profile of a plant, for example, using high-resolution mass spectrometry (HRMS) and can be used to evaluate plant stress responses, such as those due to drought. Metabolomic analysis is dependent upon the efficiency of the extraction protocol. Currently, there are two common extraction procedures widely used in metabolomic experiments, those that extract from plant tissue processed in liquid nitrogen or extraction from lyophilised plant tissues. Here, we evaluated the two using non-targeted metabolomics to show that lyophilisation can stabilise the maize (*Zea mays*) extractable metabolome, increasing throughput and efficiency of extraction as compared to the more traditional processing in liquid nitrogen. Then, we applied the lyophilisation approach to explore the effect of drought upon the maize metabolome in a non-targeted HRMS metabolomics approach. Metabolomics revealed differences in the mature maize metabolome having undergone three drought conditions imposed at two critical development stages (three-leaf stage and grain-fill stage); moreover, this difference was observed across two tissue types (kernel and inner cob/pith). It was shown that under ideal conditions, the biochemical make-up of the tissue types is different. However, under stress conditions, the stress response dominates the metabolic profile. Drought-related metabolites known from other plant systems have been identified and metabolomics has revealed potential novel drought-stress indicators in our maize system.

## 1. Introduction

Maize (*Zea mays* L.) is one of the most important food crops produced globally and is highly adaptable to different growing environments. However, drought is a major limiting factor to crop yields of maize, and climate change is threatening food security [1,2,3] due to increased periods of drought. Southern African maize has been identified as particularly at risk [1].

Metabolomics is a powerful tool for investigating the entire chemical profile of a biological sample. Metabolites and their levels are of particular interest when investigating genetic influences or environmental changes, as they can be regarded as the final response of an organism to genetic or environmental perturbations [4]. Metabolomics has previously been used to understand stress from drought [5,6,7,8,9] and salinity [10,11] in maize.

The maize cob provides the growing seed with nutrients including proteins, oils and starch [12]. Hence, lipids, amino acids, sugars and carbohydrates are expected to be the major components of a kernel extract. It has been shown that increases in the levels of osmoprotectants, such as mono-, di- and oligosaccharides, polyols and some quaternary ammonium compounds are a common adaptive measure to water stress across a range of cells and tissues; their accumulation accompanies drought stress [6]. Amines, mono-, di- and oligosaccharides and sugar alcohols can act as stabilising agents to macromolecules such as proteins; these molecules help proteins to maintain their hydration state and hence their structure [8,13,14].

Hormones are also important in plant responses to stress. For example, the plant phytohormone abscisic acid (ABA) is heavily implicated in the response to various stressors, such as drought, radiation and salt stress [15,16]. Phytohormone signalling can trigger the production of secondary metabolites whose primary function is to scavenge reactive oxygen species (ROS), which protects the plant from lipid peroxidation; for a full review see Jogawat et al. [17]. For B73, a drought-susceptible modern maize cultivar, drought stress has been shown to cause an accumulation of simple sugars and polyunsaturated fatty acids and a decrease in amines, polyamines and dipeptides, together with an increase in the accumulation of ROS and aflatoxin [9]. The accumulation of simple sugars is thought to be due to protection against oxidative stress, by osmoregulation or through ROS scavenging. Polyunsaturated fatty acids have been shown to regulate the stomatal aperture in response to drought stress [18]. An increase in ROS occurs when a plant is under drought stress and hydroperoxidation of polyunsaturated fatty acids can result in oxylipin production which can exacerbate aflatoxin contamination (see [19]).

Metabolomics approaches consider the extractable plant metabolites, and therefore the extraction protocol plays an important role in the types of molecules included in the analysis. Literature methods most often work with fresh material processed under cold conditions (for example [7]), and this can pose significant challenges. Samples are generally immediately frozen in liquid nitrogen and stored at −80 °C [20,21] to inactivate enzymatic processes. However, weighing plant material kept frozen in liquid nitrogen can be difficult due to several practical considerations such as electrostatic charging, and condensation onto frozen tissue in damp atmospheres which can cause variation in the tissue weights following drying. Lyophilising the samples prior to homogenisation removes water from the sample, which inactivates enzymes and potentially stabilises the metabolome [22]. Few studies have considered the effect of lyophilising plant material as a means to stabilise the plant metabolome [23,24] and no studies have considered its impact on metabolomic studies of an agriculturally significant crop, such as maize. 

The first objective of this study was to assess the robustness of two steps of the plant tissue extraction process. First, we compared the efficiency of lyophilisation and grinding tissue with a more traditional approach of grinding the tissue in liquid nitrogen. Next, we tested the necessity of cold room (4 °C) conditions during the solvent (70:30 methanol:water) extraction step. The effects of cold vs. room temperature extraction following lyophilisation were also considered. Our results were used to validate a safe, efficient, robust and reproducible extraction method (extraction condition experiment). The second objective was to use this method to evaluate the influence of drought stress upon mature corn cobs from plants having undergone three different watering regimes (drought condition experiment).

## 2. Results and Discussion

### 2.1. Effect of Lyophilisation on the Plant Metabolome

The effect of lyophilisation on the extractable plant metabolome was investigated using non-targeted liquid chromatography–mass spectrometry (LC–MS). Metabolites were extracted from maize cob tissue using three different extraction methods. In one set of samples, fresh tissue was ground and extracted under liquid nitrogen (LNE), another set was lyophilised, ground and extracted in the cold (4 °C) (LC), and a third group was lyophilised, ground and extracted at room temperature (LRT). The water content of samples was 69.0 ± 7.5%, as described in Table S1.

**Figure 1:**
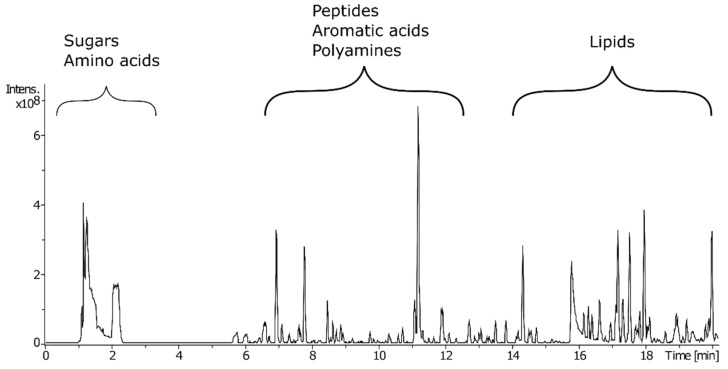
Characteristic base peak ion chromatogram for an LNE sample obtained using a T3 column coupled to high-resolution mass spectrometry (LC–HRMS) to allow the retention and separation of metabolites with a broad range of polarities.

A chromatographic separation method was designed so a wide range of feature polarities could be examined. Atlantis^®^ T3 columns are reversed-phase C18 columns that provide balanced retention of polar and hydrophobic molecules. Application of such a column using a high polar to high organic solvent concentration gradient allowed good retention and separation of metabolite classes (Figure 1). A wide range of metabolite classes was extracted from the maize tissue using methanol:water extraction; polar metabolite classes such as disaccharides and amino acids were first to elute, followed by organic acids and lipids. Figure 1 shows a characteristic chromatogram of a sample from the LNE group. Chromatograms from the LC and LRT sample groups can be seen in Figure S1. It is difficult to distinguish between samples groups based upon the base peak chromatograms, hence statistical techniques were used to assess the data.

**Figure 2:**
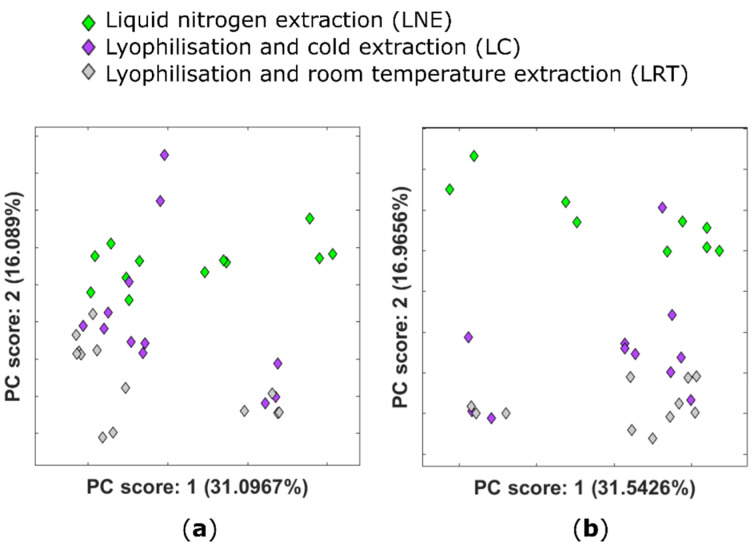
PCA plots showing the scores for the first two principal components obtained using untargeted metabolomic analysis of different extraction conditions for *Zea mays* coloured by experimental group (green, purple and grey represent LNE, LC and LRT, respectively) (**a**) for the positive ion mode; (**b**) and for the negative ion mode. The data have been scaled to unit variance and QC corrected.

To test whether there are differential effects of freeze-drying on extracted maize metabolites, an untargeted study of methanol:water-soluble metabolites was performed to identify features that change due to the different tissue extraction protocols used. Datasets of full-scan positive-ion-mode and full-scan negative-ion-mode LC–MS data, collected using a Bruker solariX Fourier-transform ion cyclotron resonance–mass spectrometer (FTICR–MS), were investigated using principal components analysis (PCA), a multivariate unsupervised statistical technique. Figure 2 shows the PCA score plots for all replicate injections of samples generated using the three different extraction conditions being compared: LNE (green diamonds), LC (purple diamonds) and LRT (grey diamonds).

Examination of PC1 for both scan polarities revealed that the majority of variance is across biological replicates. PC2 in both instances primarily shows variation due to extraction conditions. Once lyophilised, extraction in either cold or room temperature conditions had a small effect on the extractable metabolome, with more variance across the LC group than LRT. Extraction conditions therefore did not account for the majority of variance in metabolite profiles acquired in either the positive (Figure 2a) or negative ion mode (Figure 2b). 

Using liquid nitrogen or lyophilisation to stabilise the extractable metabolome of maize cob tissues seems to have limited impact on the extractable metabolome based on the PCA results. Both the extraction protocols (liquid nitrogen and lyophilisation) have been used to stabilise the extractable metabolome in previous metabolomic studies (for example [25,26]). Using liquid nitrogen to stabilise the extractable metabolome is well established, but cumbersome and requires access to specific facilities. Accurately weighing samples whilst the samples are frozen using liquid nitrogen is challenging. First, LNE relies on the assumption that water content is consistent between samples, but the moisture content of samples in this experiment had a standard deviation of ±7.5% which is considerable. Variability in the water content can be reduced by thorough grinding of samples, distributing the water crystals more evenly throughout the sample, but this can be difficult to achieve with some biological materials. Second, the electrostatic nature of frozen material and condensation from damp atmosphere on to the sample can make weighing samples difficult. Lyophilising samples prior to extraction can avoid some of these issues as dry masses can be more efficiently weighed whilst not requiring access to liquid nitrogen or a cold room. 

Very few studies have compared the effects of extraction following lyophilisation versus freezing under liquid nitrogen in plant material [24]. Studies that have been conducted have shown that freeze-drying can be used to stabilise metabolites, including plant hormones [24]. Hamid et al. [23] compared freeze-drying and oven-drying as techniques to stabilise the metabolome of seaweed, and concluded that freeze-drying yielded higher metabolite concentrations than oven-drying. However, Hamid et al. [23] did not directly compare lyophilisation to liquid nitrogen extraction. Zhu et al. [27] have shown that freeze-drying milk to produce milk powders has a limited effect on the milk metabolites; this study also showed stability in the metabolome when stored at either 4 or −20 °C, but storage at room temperature resulted in significant changes. Cheng et al. [28] suggest using lyophilised faecal samples, rather than fresh frozen samples. Our data have shown that lyophilisation can stabilise the extractable maize metabolome and resolves issues around weighing variability across tissue samples in this experiment. The effect of drying methods on the metabolome should be tested across a wider range of different biological species and tissue types. 

Investigating the effect of desiccation on the metabolome has additional value when considering archaeobotanical samples. These samples are often well preserved through natural desiccation and considering the effect desiccation has on the metabolome may offer fruitful insight into the physiological conditions under which ancient plants were grown, with obvious relevance for studies of past climates. 

### 2.2. Effect of Drought Conditions upon the Extractable Maize Metabolome

#### 2.2.1. Variation in the Extractable Metabolome of Plants Grown under Different Watering Conditions

An untargeted study of methanol:water-soluble maize metabolites was performed to identify features that undergo changes due to drought conditions and also between kernel and inner cob tissue. Two datasets of full-scan positive- and negative-ion-mode LC–MS data were investigated. Maize was grown under three different watering conditions: a well-watered control; moderate drought; and severe drought (see Section 3.1.2). Cobs were harvested at maturity in all cases.

The tissue samples were lyophilised and extracted in a cold room using the methanol:water extraction protocol. The LC extraction protocol was chosen for two reasons. The data from the extraction conditions experiment had shown that lyophilisation stabilised the extractable metabolome as effectively as liquid nitrogen extraction. The cold room extraction was chosen because at the time of sample processing, it was not yet clear what effect room temperature extraction has upon more labile molecules, and therefore the more cautious approach was taken. Considering the results of the extraction condition experiment, either LC or LRT would have been appropriate. 

**Figure 3:**
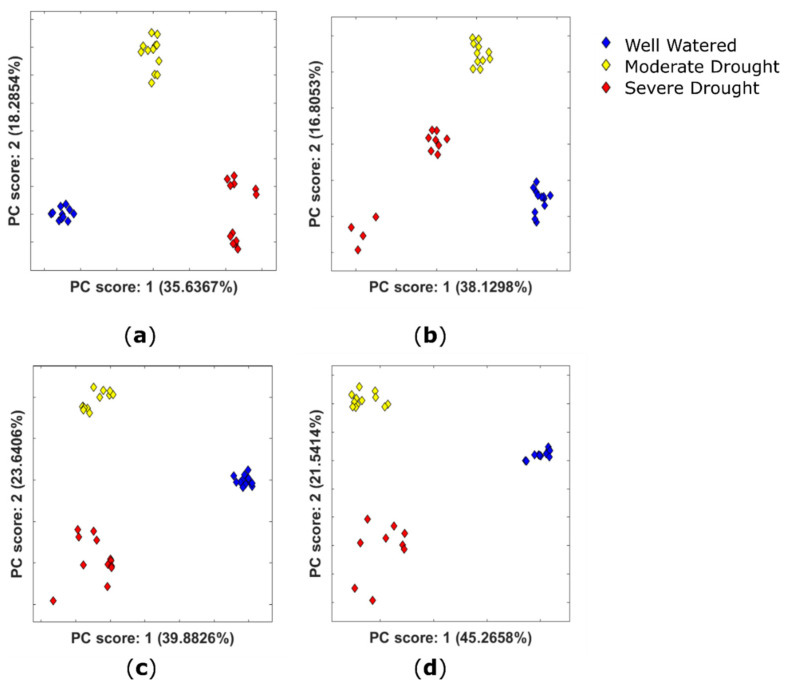
PCA plots showing the scores for the first two principal components obtained using untargeted metabolomic analysis of drought-stressed and well-watered control plants, coloured by watering conditions for *Zea mays* for (**a**) positive-ion-mode kernel extracts; (**b**) negative-ion-mode kernel extracts; (**c**) positive-ion-mode inner cob extracts and (**d**) negative-ion-mode inner cob extracts. The data have been scaled to unit variance and QC corrected.

PCA (Figure 3) shows very distinct clustering by watering conditions for extracts of both kernels and inner cob tissue. Multivariate analysis also showed a tight clustering of QC samples (not shown), which indicates that the differences in watering regimes account for the differentiation between groups rather than instrumental variation throughout the run. Figure 3 shows that separation in PC1 is characterised by differences between well-watered and drought conditions. Separation in PC2 is characterised by differences between moderate and severe drought conditions. This implies a different metabolic response to drought stress when it is applied at the three-leaf and grain-fill stages of plant development. Positive-ion-mode data show strong separation of drought conditions for both kernels and inner cob tissue, as do negative-ion-mode inner cob data. Clustering is less strong for severe drought-treated kernel negative-ion-mode data (Figure 3b, red diamonds).

#### 2.2.2. Identification of Potential Drought Biomarkers

Multivariate analysis shows that PC1 is characterised by differences between well-watered and drought conditions (Figure 3) for both tissue types for positive- and negative-ion-mode data. Therefore, the largest loadings of PC1 were investigated as potential drought biomarkers. 

**Figure 4:**
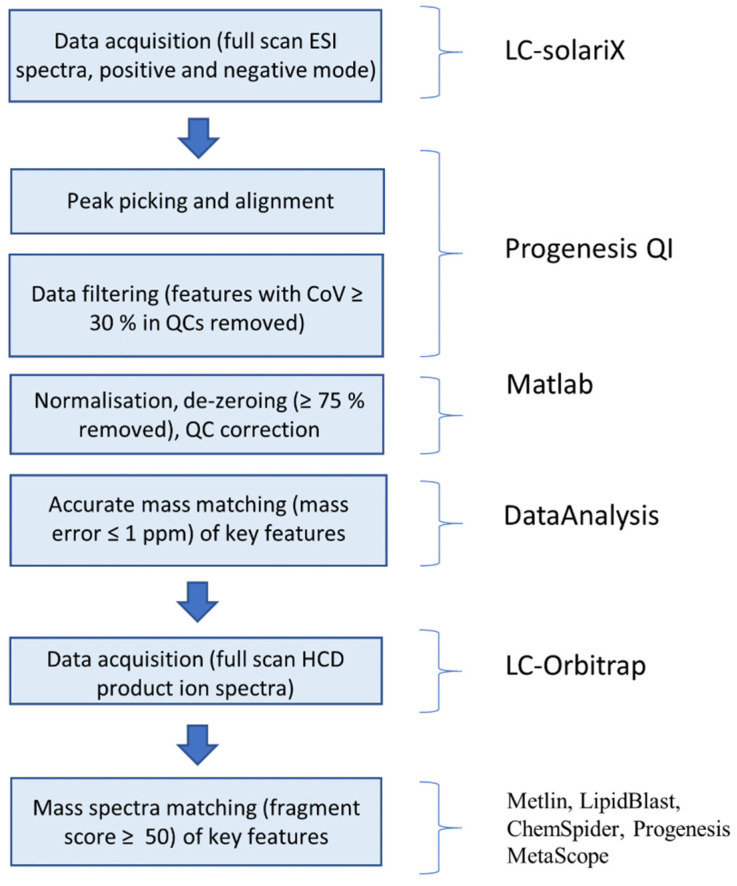
Data analysis workflow for the identification of key features in mass spectrometric data.

ANOVA tests were conducted on peak intensities of features identified as having the largest loadings in PC1. Molecular formulae were obtained from the FTICR–MS data using the ‘SmartFormula’ function of Bruker’s DataAnalysis software and corroborated using Progenesis QI. An internal lock-mass calibration was carried out on the positive-ion-mode samples in order to improve mass error (≤1% ppm). Information about all key features (*m/z*, *t*_R_, molecular formulae, ANOVA 1-way *p*-values (*p*-val)) are presented in Table S2. Where possible, higher-energy collision-induced dissociation (HCD) product ion spectra were obtained using a Thermo™ Orbitrap™ Fusion™ instrument for key features and the fragmentation patterns were interrogated against compound databases (Metlin, LipidBlast, ChemSpider, Progenesis MetaScope) in order to assign possible identifications. Table 1 displays information about key features where a possible identification has been assigned. For an identification to be accepted, the Progenesis fragmentation score was required to be greater than 50. The data analysis workflow is outlined in Figure 4. An example figure showing the relative abundances of the top four highest loadings can be found in Figure S2.

**Figure 5:**
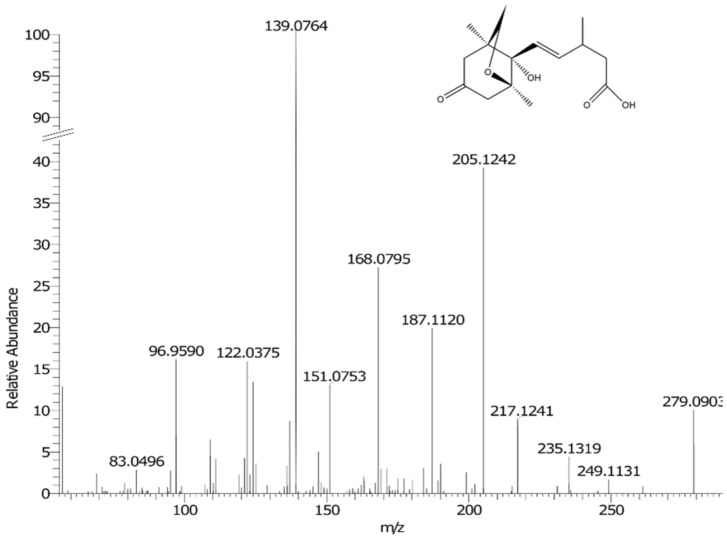
Fragmentation spectrum of *m/z* 279.124 at 9.52 min.

A feature with *m/z* 279.124 eluting at a retention time *(t*_R_*)* of 9.52 min was tentatively identified as neophaseic acid (neoPA) (product ion spectrum in Figure 5). The feature was identified by Progenesis as hydroxyabscisic acid, but following examination of Zhou et al. [29], the feature was assigned as neoPA. NeoPA is a metabolite of the phytohormone ABA which is formed following the oxidation of the 9-methyl group of ABA [29,30]. ABA is one of the most common stress signals to appear in plant organs in response to drought conditions [16,17,29,31]. ABA was detected in the drought-treated samples in this study. However, it was not included in further analysis because it had a coefficient of variation (CoV) score greater than 30%. Oxidation of ABA causes inactivation of the hormone and neo-PA is considered biologically inactive. Neo-PA has been detected in drought-stressed barley seedlings [29] and its detection here in drought-stressed maize tissue suggests a role in the abiotic stress response. 

The molecular formulae of several phospholipids (*m/z*: 480.3075, 520.3398, 522.3556, 540.3305) were also tentatively identified. Drought stress is known to have an impact on the metabolism of membrane lipids and can cause changes in the membrane lipid composition [32,33]. Phospholipids can act as signalling precursors and molecules which regulate plant growth and development, and response to environmental factors, such as drought stress [34,35].

### 2.3. Drought-Stress Response Dominates Metabolome in Kernel and Inner Cob Tissues

**Figure 6:**
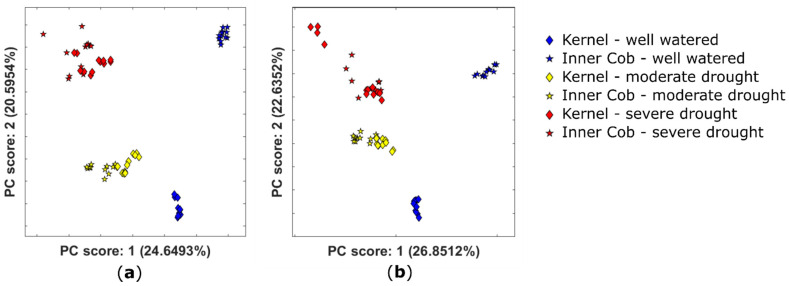
PCA plots showing the scores for the first two principal components obtained for the untargeted metabolomic analysis coloured by watering conditions for *Zea mays* for (**a**) the positive ion mode and (**b**) the negative ion mode. The data have been scaled to unit variance and QC corrected for both cases.

Two datasets of full-scan positive- and negative-ion-mode LC–FTICR–MS data were investigated to compare the metabolomes of the kernel and inner cob. The growing conditions and data handling are described in Section 2.2.1. PCA analysis (Figure 6) shows clustering of the kernel and inner cob severe drought samples (red diamonds and stars) and clustering of the kernel and inner cob moderate drought samples (yellow diamonds and stars). However, well-watered kernels and inner cob samples did not cluster together (blue diamonds and stars).

Watering conditions cause more variance in both sets of drought-treated samples (moderate and severe drought) than tissue type. During optimum growing conditions (well watered), there is a clear difference in the biochemical composition of the two tissue types as indicated by separate and distinct clusters. However, when drought stress is introduced, there is less divergence between the two tissues. This shows that the drought-stress response was dominant over tissue-specific metabolism across the two tissue types. In severely stressed plants, kernel material can be limited, but inner cob material is more likely to be available and these data show it contains useful abiotic stress signals.

## 3. Materials and Methods 

### 3.1. Plant Material Description, Cultivation and Sampling

#### 3.1.1. Extraction Condition Experiment

Four biological replicates of B73 corn were investigated. Three sample preparation conditions were compared. The inner central section of each cob was sampled in each condition (1 g). The following extraction conditions were used: liquid nitrogen extraction (LNE), the samples were ground and weighed whilst kept frozen using liquid nitrogen; lyophilised cold (LC), lyophilised maize ground at room temperature and extracted at 4 °C, and lyophilised room temperature (LRT), lyophilised maize ground at room temperature and extracted at room temperature.

For LC and LRT conditions, the samples were lyophilised and three extraction replicates were weighed (5.00 mg ± 0.09). For the LNE condition, three extraction replicates were weighed (16.12 mg ± 1.2, moisture content 68.95%).

#### 3.1.2. Drought Condition Experiment

Maize plants, B73 variety (GRIN accession number: PI 550473, developed at Iowa State University, pedigree C% and drought susceptible), were grown at the Warwick University Phytobiology Facility in a climate-controlled greenhouse. Plants were grown at 28 °C day/20 °C night in a 16-h light/8-h dark cycle with a light intensity of 230 μE m^−2^ s^−1^. Seeds were germinated in three-inch diameter pots containing peat-based soil. Soil water content was measured at regular intervals during drought stress using a Professional Soil Moisture Meter (Lutron Electronic Enterprise Co., LTD., Taipei, Taiwan) to ensure drought conditions were successfully applied. The plants were split into three groups, outlined in Table 2. Drought condition were applied during two growth stages, three-leaf and grain-fill (BBCH principal growth stage 1 and 8, respectively [36]). Drought conditions consisted of withholding water for one week, which resulted in soil water content between 0 and 1% (Table 2). Non-sterilised soil was used, and reverse osmosis water was used to water the plants. Plants were harvested for analyses when the cobs were fully ripe and were immediately frozen in liquid nitrogen and stored at −80° C prior to extraction and metabolomic analysis.

Prior to extraction, samples were lyophilised and the kernels were separated from the inner cob. For each cob, a central section of pith (1 g) was ground. For each kernel sample, 5 randomly selected kernels were ground together. Three extraction replicates were prepared (5 mg ± 0.05 mg) for each tissue and from each biological replicate (2 per sample group).

### 3.2. Metabolite Extraction Method

The plant material was extracted with methanol:water (70:30, *v*/*v*, 100 µL). The extraction process consisted of shaking at 500 rpm for 30 min in dark conditions, followed by centrifugation at 15,200 rpm for 10 min. The supernatant was transferred to a clean tube, dried using a vacuum concentrator (whilst kept cool) and reconstituted in methanol:water (70:30, *v/v*, 100 μL) and subsequently analysed using HPLC–FTICR–MS. For the LNE and LC conditions, the samples were kept at 4 °C during extraction and for the LRT condition, the samples were extracted at room temperature. All samples were extracted in the cold room for the drought conditions experiment.

### 3.3. Data Collection 

In the extraction condition experiment, three extraction replicates were taken for each condition and two technical replicates were taken for each extract. For analysis by LC–MS, the sample injections were separated into three semi-randomised batches containing one technical replicate from each extract. Quality controls (QCs) composed of a pooled aliquot of 5 uL from each sample were injected at the start of the experiment (20 injections) to equilibrate the column. A QC and a blank were injected after every five sample injections.

For the drought condition experiment, three extraction replicates were taken for each condition and two technical replicates were taken for each sample. Data collection was the same as that described in the paragraph above.

### 3.4. LC–MS-Based Analysis of Metabolome

#### 3.4.1. Liquid Chromatography Separation Method

HPLC–MS was carried out using an Agilent 1200 HPLC fitted with an Atlantis^®^ T3 column (Waters, cortecs 2.7 µm, 3 × 150 mm); the column temperature was maintained at 25 °C. The HPLC was coupled to an electrospray FT-ICR mass spectrometer (Bruker solariX XR 9.4T). The mobile phase was composed of water (A) and acetonitrile (B) both with 0.1% (*v/v*) formic acid, and the following gradient was used: 5% B increasing linearly to 95% over 22 min and held for 2 min. B was then returned to 5% over 0.33 min and held for 5.33 min to allow column equilibration. The flow rate was 300 µL/min and the injection volume was 5 µL.

#### 3.4.2. Mass Spectrometry Methods

Positive ion mode: The LC methods are as described in Section 3.4.1. The Bruker solariX XR 9.4T was operated over the *m/z* range 57.75–2000.00 with an electrospray ionisation source. Analytes were detected in the positive ion mode using the following MS parameters: the dry gas flow was 7.0 L/min; the dry gas temperature was 200 °C, the source voltage was 4000 V and the nebuliser gas pressure was 2.0 bar. 

Negative ion mode: The LC methods are as described in Section 3.4.1. The Bruker solariX XR 9.4T was operated in the negative ion mode over the *m/z* range 57.75–2000.00 with an electrospray ionisation source. Analytes were detected in the negative ion mode using the following MS parameters: dry gas flow was 7.0 L/min; the dry gas temperature was 200 °C and the source voltage was 4000 V. To optimise ionisation, the nebuliser gas pressure was 2.0 bar (0–6 min), 1.5 bar (6–12 min), 1.3 bar (12–21 min), 1.0 (21–25 min), 1.5 (15–26 min), 1.8 (26–27.5 min) and 2.0 bar (27.5–30 min).

### 3.5. LC–MS–MS

#### Mass Spectrometry Methods

The LC methods are as described in Section 3.4.1. The Orbitrap™ Fusion™ MS was operated in the positive ion mode and the negative ion mode with the following parameters. The *m/z* range was 85–1000 with an electrospray ionisation source. Analytes were detected in the positive ion mode using the following MS parameters: the ion transfer temperature was 325 °C; the vaporiser temperature was 350 °C; the sheath gas was 50 (arb); the aux gas was (arb) 10; time between master scans was 1 s; the isolation window was 1.6; the collisional energy was stepped and the HCD collision energies (%) were 20, 35, and 60. 

### 3.6. Data Handling and Analysis

For the extraction conditions experiment, Progenesis QI (Waters, Milford, MA, USA), a dedicated software package for processing LC–MS data, was used for alignment and peak picking. Bruker .d files were imported into Progenesis in centroid mode. The runs were aligned and alignment was accepted if within 80% of the reference run. Experimental groups were used as described in Section 3.1.1.

At the peak picking stage, the following parameters were set to reduce the volume of data to allow the computer to process it. Peaks with a *t*_R_ of less than 1 min and peaks with a *t*_R_ greater than 21 min were excluded on the basis of poor separation. A minimum peak width of 0.05 min was set. The list of expected charge-bearing species [M]^+^, [M + H]^+^, [M + 2H]^2+^, [2M + H]^+^, [M + K]^+^ and [M + Na]^+^ for the positive ion mode and [M − H]^−^, [M + Cl]^−^, [M − 2H]^2−^, [M + FA − H]^−^, [2M − H]^−^ and [M − H_2_O − H]^−^ for the negative ion mode were chosen for consideration, as they would arise from the same compound. In the raw data 23,178 and 16,044 features were identified in the positive and negative ion modes, respectively. Features with a coefficient of variation greater than 30% in QC injections were excluded from further analysis.

The settings for Progenesis QI were the same for the drought experiment as in the extraction condition experiment, unless stated. Experimental groups were used as outlined in Section 3.1.2. Peaks with a *t*_R_ of less than 1 min and peaks with a *t*_R_ greater than 21 min were excluded on the basis of poor separation. In the raw data, 4912 and 11,531 features were identified in the positive and negative ion modes, respectively.

### 3.7. Statistical Analysis

For the extraction condition experiment, statistical tests were conducted using the programming environment MATLAB (Mathworks, Natick, MA, USA). The data were normalised using the total ion count, de-zeroed at a rate of 75% and QC corrected. 8483 and 10,715 features were included in the analysis (positive and negative ion modes, respectively). Data were scaled for PCA. For each feature, one-way analysis of variance (ANOVA) was used to test the statistical significance of differences between groups with Benjamini–Hochberg adjustment to correct for multiple testing.

Statistical tests were carried out as described in the paragraph above for the drought conditions experiment. Following data filtering, 1105 and 2191 features were included in the positive and negative analyses, respectively.

## 4. Conclusions

This study has shown that lyophilising plant material is an effective way to stabilise the extractable plant metabolome in maize and is as effective as extracting under liquid nitrogen. Lyophilisation is thus an adequate alternative to the more challenging, resource-intensive and potentially hazardous liquid nitrogen processing techniques. This observation has the potential to improve sample processing efficiencies which can be a bottle neck in metabolomics workflows. However, the effect of desiccation on labile molecules such as phytohormones and the utility of cold room extractions following lyophilisation require further study. The lyophilisation extraction technique also has useful applications to archaeobotanical studies where archaeological plant tissues have been naturally preserved via desiccation.

Divergence in the metabolomic profiles of the experimentally drought-stressed maize studied here occurs with the onset of drought stress at the three-leaf stage. Drought stress at the grain-fill growth stage indicated a further divergence from the metabolome of well-watered plants. Differences in response to drought stress were also observed in two plant tissues, kernels and inner cob. It was shown that the drought-stress response was dominant across both tissue types and that inner cob material can be a useful source of information when no kernel data can be obtained. Non-targeted metabolomic profiling is clearly a powerful tool to better understand the biochemical mechanisms maize employs to cope with drought stress.

## Figures and Tables

**Table 1:** Summary of data on all possible identifications of drought-related biomarkers of maize. Possible identifications were assigned using compound databases (Metlin, LipidBlast, ChemSpider, Progenesis MetaScope). * Indicates the data were scaled. The *p*-val between the well-watered and severe drought conditions is quoted.

Tissue Type	Scan Mode	*m/z*	*t*_R_	Molecular Species	MF	ppm	*p*-val	Possible Identification	Fragmentation Score
K	N *	279.1238	9.52	[M − H]^−^	C_15_H_20_O_5_	−0.04	9.60 × 10^−15^	Hydroxyabscisic acid or Neophaseic acid (neoPA)	50.1 *
K	N *	143.0349	3.88 and 3.89	[M − H]^−^	C_6_H_8_O_4_	0.30	1.78 × 10^−14^	Methyl itaconate	58.5
K	P *	480.3075	18.87	[M + H]^+^	C_23_H_46_NO_7_P	−0.21	1.19 × 10^−22^	PE(18:1/0:0)	55.1
K	P	520.3398	17.75	[M + H]^+^	C_26_H_50_NO_7_P	−0.10	1.28 × 10^−18^	PC(18:2/0:0)	88.8
K	P	522.3556	18.99	[M + H]^+^	C_26_H_52_NO_7_P	−0.46	2.42 × 10^−16^	PC(18:1/0:0)	78.4
IC	N	540.3305	18.51	[M + FA − H]^−^	C_24_H_50_NO_7_P	0.37	1.81 × 10^−8^	Lysolecithin	63.5

* Fragmentation score is given for hydroxyabscisic acid.

**Table 2:** Table showing the watering conditions for each plant growth condition group.

Condition Group	Details
Well watered	Samples were well watered for the duration of the experiment
Moderate drought	Drought stressed for two one-week periods. The first week starting at the three-leaf stage (approximately 4 weeks after seeding). The plants were watered for a further week and then droughted for another one-week period
Severe drought	Drought stressed as described for moderate drought conditions and then for a further two weeks during grain-fill (approximately 14 weeks after seeding)

## Data Availability

The data presented in this study are openly available in Zenodo at 10.5281/zenodo.5060311.

## Supplementary Materials

The following are available online at https://www.mdpi.com/article/10.3390/metabo11070438/s1, Figure S1: Characteristic base peak ion chromatograms for LNE, LC and LRT samples obtained using a T3 column coupled to high-resolution mass spectrometry (LC–HRMS) to allow the retention and separation of metabolites with a broad range of polarities, Figure S2: Figure showing relative abundances of *m/z* features (a) 279.1238 (b) 143.0349 (c) 480.3075 and (d) 520.3398. Blue, yellow and red diamonds represent well-watered, moderate drought and severe drought conditions, respectively. ANOVA *p*-vals are indicated, Table S1: Table showing the moisture content of the maize used in the extraction condition experiment, Table S2: Summary of data on all possible drought-related biomarkers of maize. Possible identifications were assigned using compound databases (Metlin, LipidBlast, ChemSpider, Progenesis MetaScope *Indicates the data were scaled. The adjusted ANOVA *p*-val is quoted.
