# Supplementary material for: Metabolomic Approaches to Studying the Response to Drought Stress in Corn (Zea mays) Cobs
Source: Metabolites. 2021 Jul 3;11(7):438. doi: 10.3390/metabo11070438 (PMC8305929; doi:10.3390/metabo11070438)
Supplement: Supplementary file 1 [file metabolites-11-00438-s001.zip › metabolites-1266371-supplementary.pdf]

metabolites

Supplementary Materials:

The link to the RAW data files will be made available prior to publication.

Figure S1. Characteristic base peak ion chromatogram for (a) LNE (b) LC and (C) LRT samples obtained using a T3 column coupled to high resolution mass spectrometry (LC-HRMS) to allow the retention and separation of metabolites with a broad range of polarities.

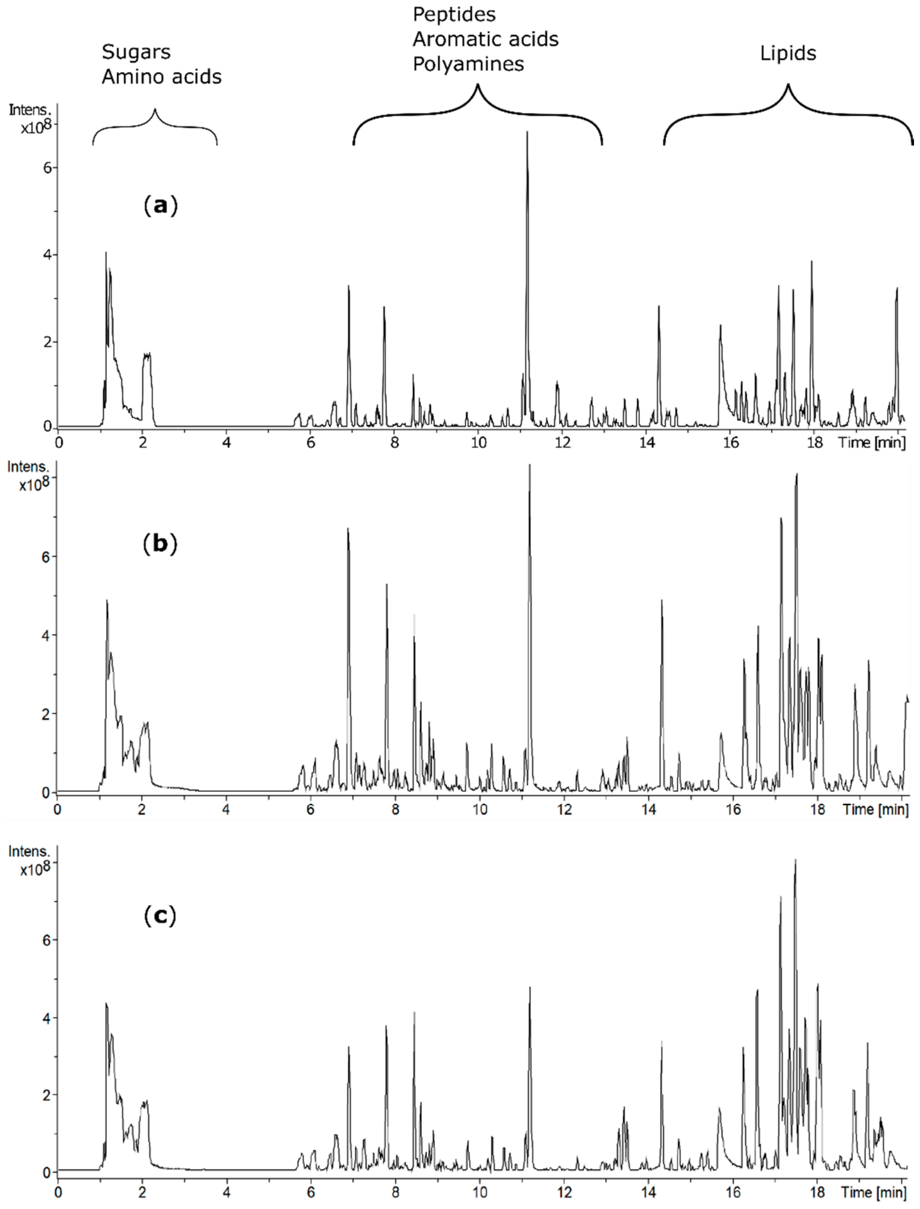

Figure S2. Figure showing relative abundances of *m/z* features (**a**) 279.1238 (**b**) 143.0349 (**c**) 480.3075 and (**d**) 520.3398. Blue, yellow and red diamonds represent well-watered, moderate drought and severe drought conditions respectively. ANOVA p-vals are indicated.

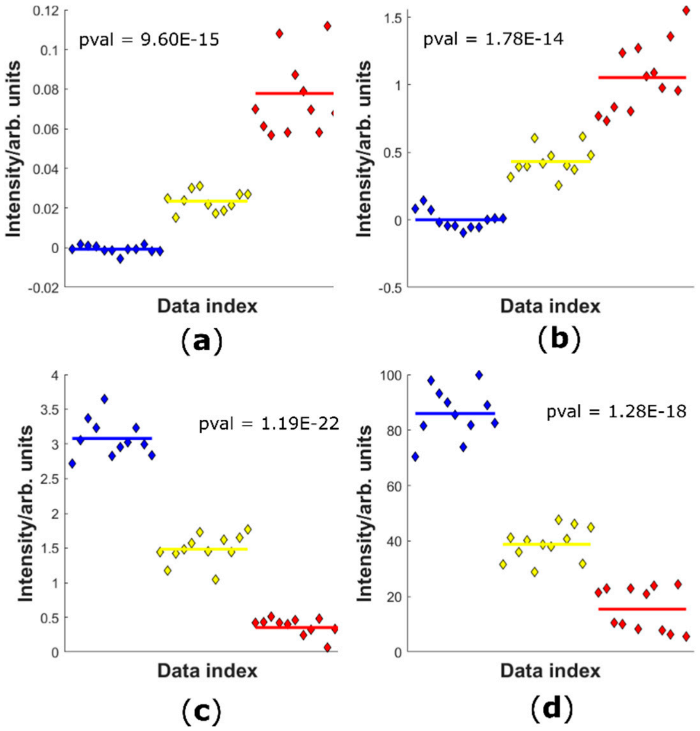

Table S1. Table showing the moisture content of the maize used in the extraction condition experiment.

Sample	Fresh weight (g)	Dry weight (g)	Moisture content (%)
B73I	1.199	0.237	80
B73H	1.748	0.603	66
B73G	1.798	0.627	65
B73J	1.923	0.687	64

Table S2. Summary of data on all possible drought related biomarkers of maize. Possible identifications were assigned using compound databases (Metlin, LipidBlast, ChemSpider, Progenesis MetaScope *Indicates the data were scaled. The adjusted ANOVA p-val is quoted.

Tissue Type	Scan Mode	*m/z*	*t*R	Molecular Species	MF	ppm	p-val	Possible Identification	Fragmentation Score
K	N*	279.1238	9.52	$[M-H]^-$	$C_{15}H_{20}O_5$	-0.04	9.60E-15	Neophaseic Acid (neoPA)	50.1
K	N*	143.0349	3.88 and 3.89	$[M-H]^-$	$C_6H_8O_4$	0.3	1.78E-14	Methyl itaconate	58.5
K	N*	129.0193	2.05	$[M-H_2O-H]^-$	$C_5H_8O_5$	0.4	6.73E-14	Citramalic acid	29.4
K	N*	345.0827	2.8	$[M-H]^-$	$C_{14}H_{18}O_{10}$	0.02	3.71E-9	No fragmentation information	-
K	N	423.2517	21.18	$[M+FA-H]^-$	$C_{19}H_{39}O_5P$	0.05	1.25E-10	No fragmentation information	-
K	N	447.2516	19.95	$[M-H_2O-H]$	$C_{22}H_{43}O_8P$	-0.32	8.07E-14	-	-
K	N	375.0570	3.88	$[M-H]^-$	$C_{14}H_{16}O_{12}$	-0.24	1.85E-13	-	-
K	N	632.2858	11.88	$[M-H]^-$	$C_{29}H_{48}NO_{12}P$	-1.92	2.301E-3	-	-
K	N	735.2141	7.19	$[M-H]^-$	$C_{34}H_{40}O_{18}$	0.09	3.19E-18	No fragmentation information	-
K	P*	480.3075	18.87	$[M+H]^+$	$C_{23}H_{46}NO_7P$	-0.21	1.19E-22	PE(18:1/0:0)	55.1
K	P*	260.1700	17.77	$[M+2H]^{2+}$	$C_{23}H_{46}N_6O_5S$	-0.5	4.86E-18	No fragmentation information	-
K	P	520.3398	17.75	$[M+H]^+$	$C_{26}H_{50}NO_7P$	-0.1	1.28E-18	PC(18:2/0:0)	88.8
K	P	634.3008	11.80	$[M+H]^+$	$C_{36}H_{43}NO_9$	-0.35	4.55E-4	-	-
K	P	522.3556	18.99	$[M+H]^+$	$C_{26}H_{52}NO_7P$	-0.42	2.42E-16	PC(18:1/0:0)	78.4
K	P	478.2929	17.66	$[M+H]^+$	$C_{23}H_{44}NO_7P$	-0.26	9.06E-16	No fragmentation information	-

K	P	493.2810	8.07	$[M+H]^+$	$C_{28}H_{36}N_4O_4$	-0.09	2.01E-5	No fragmentation information	-
IC	N*	538.1745	8.37	$[M-H]^-$	$C_{24}H_{25}N_7O_8$	1.0	8.46E-26	-	-
IC	N*	559.1068	9.91	$[M-H]^-$	$C_{19}H_{29}O_{17}P$	-0.7	1.89E-14	-	-
**IC**	N*	491.1194	9.89	$[M-H]^-$	$C_{23}H_{23}O_{12}$	0.3	2.81E-23	Aurantio-obtusin β-D-glucoside	35.4
IC	N	423.2514	21.18	$[M+FA-H]^-$	$C_{19}H_{39}O_5P$	-0.04	7.29E-16	No fragment info	-
IC	N	447.2517	19.95	$[M-H_2O-H]$	$C_{22}H_{43}O_8P$	-0.4	1.18E-5	-	-
IC	N	483.2729	19.32	$[M-H]^-$	$C_{22}H_{45}O_9P$	-0.91	3.04E-14	-	-
IC	N	571.2889	18.00	$[M+FA-H]^-$	$C_{24}H_{47}O_{10}P$	-0.05	8.75E-9	-	-
IC	N	540.3305	18.51	$[M+FA-H]^-$	$C_{24}H_{50}NO_7P$	0.37	1.81E-8	Lysolecithin	63.5
IC	P*	370.1131	6.54	$[M+H]^+$	$C_{16}H_{19}NO_9$	0.46	6.59E-28	-	-
IC	P*	527.1550	12.71	$[M+H]^+$	$C_{27}H_{26}O_{11}$	-0.35	8.17E-31	No fragment info	-
IC	P*	493.1340	9.84	$[M+H]^+$	$C_{23}H_{24}O_{12}$	0.02	1.56E-28	No fragment info	-
IC	P*	540.1900	8.31	$[M+H]^+$	$C_{27}H_{30}N_3O_7P$	-0.5	4.23E-29	No fragment info	-
IC	P*	527.1537	13.05	$[M+H]^+$,	$C_{27}H_{26}O_{11}$	0.2	8.17E-31	-	-
IC	P	441.2022	10.95	$[M+H]^+$	$C_{24}H_{28}N_2O_6$	-0.31	3.60E-19	No fragment info	-
IC	P	903.3771	10.97	$[M+H]^+$	$C_{49}H_{58}O_{16}$	0.2	3.72E-24	-	-
IC	P	265.1547	6.83	$[M+H]^+$	$C_{14}H_{20}N_2O_3$	0.04	1.10E-28	Feruloyl putrescine	43.2
IC	P	411.1914	10.84	$[M+H]^+$	$C_{23}H_{26}N_2O_5$	0.1	3.60E-19	No fragment info	-
